# Comparative Evaluation of Deciduous and Permanent Coronal Caries Using Polarizing Light Microscopy and Scanning Electron Microscopy

**DOI:** 10.1155/tswj/4431399

**Published:** 2025-01-07

**Authors:** Nithya Annie Thomas, Sreena T., Charisma Thimmaiah, Pinky Varghese, Nimmy Sabu, Pretty Prince P., Athira Kattachirakunnel Sasi

**Affiliations:** ^1^Department of Pediatric and Preventive Dentistry, Manipal College of Dental Sciences, Manipal, Manipal Academy of Higher Education, Manipal 576104, Karnataka, India; ^2^Department of Oral Pathology, Mar Baselios Dental College, Thangalam, Kothamangalam 686691, Kerala, India; ^3^Department of Pediatric and Preventive Dentistry, Manipal College of Dental Sciences, Mangalore, Manipal Academy of Higher Education, Manipal 576104, Karnataka, India; ^4^Department of Prosthodontics, Indira Gandhi Institute of Dental Sciences, Nellikuzhi, Kothamangalam 686691, Kerala, India; ^5^Department of Paediatric and Preventive Dentistry, Mar Baselios Dental College, Thangalam, Kothamangalam 686691, Kerala, India; ^6^Department of Oral Pathology, Indira Gandhi Institute of Dental Sciences, Nellikuzhi, Kothamangalam 686691, Kerala, India

**Keywords:** deciduous carious teeth, permanent carious teeth, polarizing microscopy, scanning electron microscopy

## Abstract

**Background:** Dental caries causes mineral loss and organic damage to teeth. Understanding caries and dentin pulp reactions is crucial for effective caries management strategies. There is a lack of knowledge regarding the microscopic and ultramicroscopic changes that occur during caries destruction and reactive changes. This study used polarizing and scanning electron microscopy to compare deciduous and permanent coronal caries.

**Materials and Methods:** The study included 30 teeth, comprising 15 primary and permanent teeth, all with coronal caries. They were also compared with 10 (5 each) noncarious primary and permanent teeth. The teeth were examined using polarizing and scanning electron microscopy to study enamel and dentin destruction, reactive dentin formation, peritubular dentin destruction, and bacterial colonization.

**Results:** Deciduous teeth had more enamel and dentin destruction for coronal caries than permanent teeth in our study. The permanent teeth had more reactive dentin formation than primary teeth. Peritubular dentine alteration was increased in deciduous teeth, and bacterial presence on dentin was higher in permanent teeth under scanning electron microscope.

**Conclusion:** Our findings suggest that caries destruction is more prevalent in deciduous teeth, and reactive response is more effective in permanent teeth affected by caries. These findings reflect the structural durability of the mineralized tissues and prompt reactive response of the dentin pulp complex of permanent teeth compared to deciduous teeth. Our research highlights the importance of identifying and treating decay in primary teeth at an early stage.

## 1. Introduction

Dental caries is an irrevocable microbiologic infection of the calcified dental structures, expressed by demineralizing the inorganic segment and destroying the organic structure [1–3]. The equilibrium between demineralization and remineralization processes determines the state of tooth enamel. When this balance is disrupted, caries occur [4]. In 2010, 2.4 billion adults with permanent teeth and 621 million children with primary teeth had untreated caries. It was the most common disease among adults and the 10th among children [5]. There is a notable difference in structure and composition between the primary and permanent teeth. The composition of primary teeth enamel differs from permanent teeth enamel in terms of thickness, porosity, and mineralization. It has more carbonate and less phosphorous and calcium phosphate [6–8]. Most permanent teeth dentin has “*s*” shaped curvature, while most deciduous teeth dentin has a straight course, allowing an easy microbial progression [4]. The density of tubules in primary tooth dentin is lower than that of permanent tooth dentin [9]. The genre of microflora in the permanent and deciduous teeth is also different. Understanding the difference in the nature of caries destruction and dentin pulp complex reaction between the permanent and deciduous teeth is necessary for designing clinical strategies for caries management [10–12]. There is a deficit of knowledge on understanding caries destruction and reactive alterations accompanying them at microscopic and ultramicroscopic levels.

Various techniques have been employed to investigate dental tissues, including microradiography, energy-dispersive x-ray spectroscopy, x-ray diffraction, scanning electron microscopy (SEM), and microcomputed tomography-obtaining high-resolution information on morphological features/elemental composition from samples of interest [10, 13]. The objective of this study was to evaluate and compare the destruction of enamel prism, dentinal tubules, reactive dentin formation using polarizing microscopy in deciduous and permanent teeth with occlusal dentinal caries and to evaluate and compare peritubular dentine alteration and bacterial presence on dentin using a SEM in deciduous and permanent teeth with occlusal dentinal caries.

## 2. Methodology

This study was conducted by the Department of Oral and Maxillofacial Pathology at Mar Baselios Dental College, Kothamangalam. The differences in microscopic and ultramicroscopic changes between deciduous and permanent teeth affected by coronal caries, focusing on variations in enamel and dentin destruction, the formation of reactive dentin, alterations in peritubular dentin, and bacterial colonization, were investigated using polarizing and SEM. Patients with carious teeth indicated for extraction reported in the Department of Oral & Maxillofacial Surgery and Pediatric & Preventive Dentistry were included in the study after approval by the Institutional Ethical Committee (IEC/23/2012/MBDC). The sample size was calculated based on the study by Chowdhary and Subba Reddy [13] This calculation was based on the assumption that the confidence interval is 95%; power is 80%, level of significance was set at 0.001 a true difference between treatments, adjusted to 2 units. This was based on the calculations by SPSS software Version 20.

The study was conducted on a total of 40 extracted teeth, including 15 permanent molars and 15 deciduous molars. Gross caries destruction extending up to middle 1/3^rd^ of crown and patients with occlusal dentinal caries on permanent and deciduous molars with minimal pulp exposure with age group between 13 and 20 years for permanent molars and between 7 and 12 for deciduous molars were included in group I and group II, respectively. In group III, 10 teeth were selected with 5 permanent and 5 deciduous molars with no caries lesions indicated for serial extractions and exfoliated teeth and permanent third molars indicated for extractions within the age group of 9–70 years [14, 15].

Following extraction, the specimens were placed in a solution of 0.9% saline and 0.5% thymol (1:1) at 4°C for no more than 1 month before the experiment began [16]. The teeth that were chosen for the procedure were first cleaned using deionized water. After that, any remnants of soft tissue were separated using a scalpel. Finally, they were sliced longitudinally into two portions with a diamond disc (BesQual, New York, NY) mounted on a low-speed motor (Brasseler, Savannah, GA) [17]. In the process described, one of the longitudinal sections was reduced by half using a lathe, with intermittent cooling facilitated by water. The remaining longitudinal sections were reserved for the purpose of SEM study. The section was then finely reduced, and its surface was polished. Subsequently, the teeth were washed and placed in distilled water for a duration of 24 h for storage. Throughout the sample preparation phase, the teeth were stored in fresh, distilled water at 4°C [18]. Each section was examined under the polarizing microscope at 4x, 10x, and 40x magnifications in air, water, and quinoline. The quinoline imbibed sections were used to study the length and areas of incipient caries in enamel and enamel lesions near DEJ of both deciduous and permanent teeth. The water-imbibed sections analyzed caries destruction's length, breadth, and area. The air-dried sections were used to analyze the length and area of sclerotic dentin formation in deciduous and permanent teeth with occlusal caries. The photomicrograph was taken under 4x, 10x, and 40x. The images were subjected to analysis using the “lynbioluxauto” software.

The sections were air-dried to observe areas of destruction, water-imbibed to monitor the lesion body, and quinoline-imbibed to observe incipient caries. The other half of the longitudinal section was studied under SEM. From the longitudinal section, the root fragment was reduced in the lathe with periodic cooling with water, and the crown section was reduced to approximately 2 mm. After reduction, the sections were finely polished with coarse-grain emery paper, followed by fine-grain emery paper. The samples were then washed in distilled water for 5 min, followed by complete dehydration through a graded alcohol series (70%, 80%, 90%, and 100% alcohol) and kept in an incubator at 37 degrees Celsius for 24 h [13–15]. The specimen was then stored in plastic boxes with silica gel till observation under the electron microscope. For electron microscopic observation, the specimens were mounted on SEM stubs. Mounted specimens were sputter-coated, and samples were investigated using a SEM operating at an accelerating voltage of 20 kV. Peritubular dentine formation, dentinal tubular destruction, and bacterial presence within the tubule in deciduous and permanent teeth with occlusal dentinal caries were studied in the specimen. The area of each specimen was scanned to obtain tubules in a circular cross-section. For qualitative analysis, representative photomicrographs were obtained from selected fields at varying magnifications (800x, 1200x, 1500x, 2000x, 2500x, 3000 x, and 6000x). The pictures were collected for further interpretation. Based on the ultramicroscopic examination, the peritubular dentin destruction in deciduous and permanent teeth with coronal caries was graded into mild, moderate, and severe. Based on the ultramicroscopic examination, the bacterial concentration in deciduous and permanent teeth with coronal caries was graded into mild, moderate, and severe.

The data were recorded and evaluated using the Statistical Package for Social Sciences Version 16 (SPSS V16). As summary statistics, percentages and mean with standard deviation were calculated for the parameters. The median and interquartile range were calculated as summary statistics for incipient enamel caries at DEJ. Means of incipient enamel lesions, sclerotic dentine, and dentin destruction between permanent and deciduous teeth were compared using independent samples *t*-test. The area of the incipient enamel lesions at DEJ was compared between permanent and deciduous teeth using the Mann–Whitney test. Grades of peritubular dentine destruction and bacterial concentrations obtained by SEM studies were compared between permanent and deciduous teeth using Mann–Whitney test. A *p*-value lower than 0.05 was deemed statistically significant.

## 3. Results

The study samples included permanent teeth with coronal caries (*n* = 15) and deciduous teeth with coronal caries (*n* = 15). When the collected specimens were assessed, eight permanent teeth with coronal caries (*n* = 8), nine deciduous teeth with coronal caries (*n* = 9), and all collected noncarious (permanent teeth *n* = 5 and deciduous teeth *n* = 5) teeth fulfilled the requirement for carrying out SEM study.

**Figure 1:**
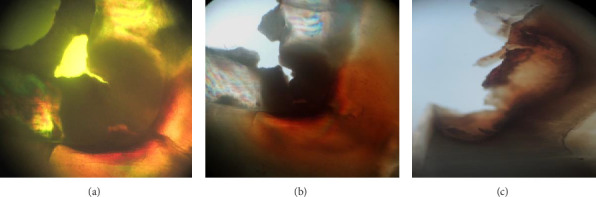
(a) Polarizing microscopic view of coronal caries imbibed in quinoline and (b) under water and (c) under air.

When the incipient enamel caries were compared between the deciduous (Group I) and permanent teeth (Group II) with coronal caries under quinoline employing a polarizing microscope, there was a rise in the average area of incipient enamel caries in deciduous teeth when compared with permanent teeth but statistically insignificant (*p*=0.651; Figure 1(a), Tables 1 and 2).

When incipient caries approximating the DEJ were compared between the deciduous (Group I) and permanent teeth (Group II) under quinoline using a polarizing microscope, only 20% of permanent and deciduous teeth revealed incipient caries near DEJ. There was barely a negligible surge in the average area of caries of permanent teeth. This difference was statistically insignificant (*p*=0.2, Table 3).

When the dentin obliteration was compared between deciduous and permanent teeth with coronal caries using a polarizing microscope underwater, there was a significant increase in dentin destruction in deciduous teeth in comparison to permanent teeth (*p* < 0.001; Figure 1(b); Tables 1 and 4).

When the sclerotic dentin emergence was compared between the deciduous and permanent teeth with coronal caries using polarizing microscope under air, there was a significant surge in the area of sclerotic dentin formation in permanent teeth compared to deciduous teeth (*p* < .001; Figure 1(c); Tables 1 and 4).

**Figure 2:**
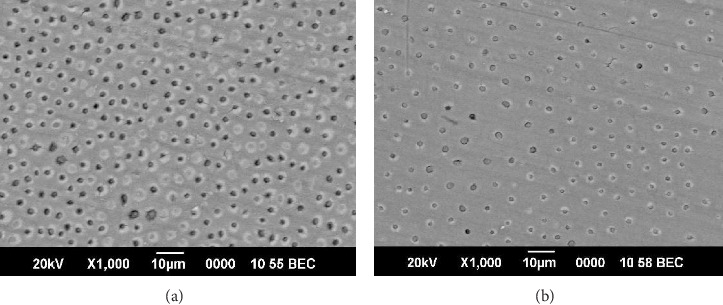
(a) Scanning electron microscopic view of normal permanent tooth. (b) Scanning electron microscopic view of normal deciduous tooth.

**Figure 3:**
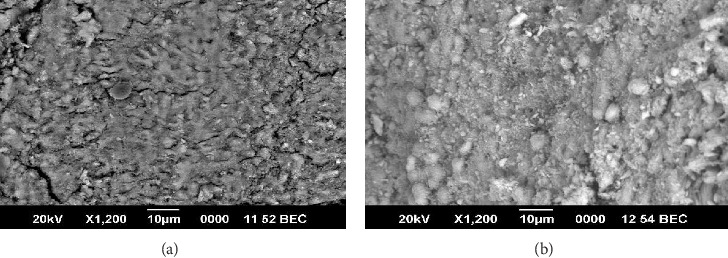
(a) Scanning electron microscopic view of peritubular destruction in permanent tooth with coronal caries. (b) Scanning electron microscopic view of peritubular destruction in deciduous teeth with coronal caries.

Peritubular dentin destruction was noticeable in the carious permanent and deciduous teeth when they were compared with noncarious deciduous and permanent teeth using SEM (Figures 2(a) and 2(b)). In Group I deciduous teeth, 55% had severe destruction and 44% had moderate destruction. In permanent teeth, 62% had mild destruction and 37% had moderate destruction. There was a significant increase in the peritubular destruction in Group I compared to Group II (*p* < 0.002; Figures 3(a) and 3(a); Table 5).

**Figure 4:**
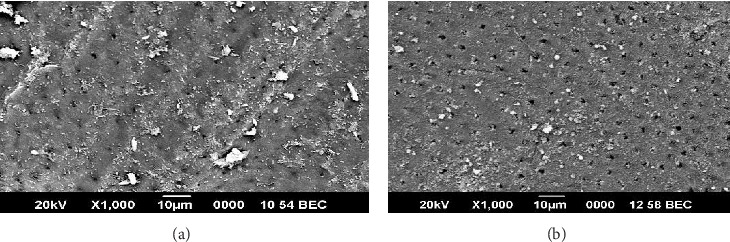
(a) Scanning electron microscopic view of bacterial colonization in permanent teeth with coronal caries. (b) Scanning electron microscopic view of bacterial concentration in deciduous teeth with coronal caries.

Microbial colonization was evident in the carious permanent and deciduous teeth when they were compared with noncarious deciduous and permanent teeth using SEM (Figures 2(a) and 2(b)). In Group I deciduous teeth, 44% had mild bacterial concentration and 11% had moderate bacterial concentration. In permanent teeth, 12% had mild bacterial accumulation, 37% had moderate bacterial concentration, and 37% had acute bacterial colonization. There was a significant increase in bacterial concentration in Group II compared to Group I (*p* < 0.015; Figures 4(a) and 4(b); Table 5).

## 4. Discussion

SEM is a routinely practiced technique to assess the surface characteristics of enamel qualitatively [19]. Therefore, the use of SEM was appropriate for studying both primary and permanent teeth in this research. Lee, Shey, and Cobb in their study on ultrastructural variations in surface properties of white-spot lesions found distinct surface and subsurface zones after imbibition in water. The surface and subsurface zones merged and disappeared when sections were imbibed with quinoline [20].

In our study, when the average area of incipient enamel caries was compared between the deciduous and permanent teeth with coronal caries, there was an increase in the average area in deciduous teeth when compared with permanent teeth but without any statistical significance (*p*0.651; Tables 1 and 2). When the incipient enamel caries near DEJ were compared between the deciduous and permanent teeth, only 20% of permanent and deciduous teeth showed incipient enamel caries near DEJ. There was only a marginal increase in the average area of incipient enamel caries at DEJ in permanent teeth. This change was statistically insignificant (*p* 0.2: Table 3: Figure 1(a)). Low, Duraman, and Davies did a study comparing the microstructure of human adult and baby teeth. He found human primary and permanent teeth exhibited distinct similarities like hydroxyapatite being the dominant state with the enamel rods running approximately perpendicular from DEJ towards the tooth surface [21]. These similarities in the structure of enamel in deciduous and permanent teeth could be the reason for the lack of statistically significant difference for enamel carious lesions between the two groups. In a study by Singh et al. among 400 school children aged 6-12 years in Faridabad city, found an increased prevalence of dentinal caries in primary teeth (95.5%) than in permanent teeth (47.3%) [22]. When dentin destruction was compared between deciduous and permanent teeth underwater, there was a significant rise in destruction in primary teeth compared to permanent teeth (*p* < 0.001; Tables 1 and 4; Figure 1(b)). In a study conducted by Chowdary and Subba Reddy, primary and permanent molars were compared using a polarizing microscope. The researchers found that most deciduous teeth dentin had a straight course, while most permanent teeth dentin had an “*S*”-shaped curvature [13]. The lower level of mineralization and thin enamel of deciduous teeth could contribute to their whiter appearance compared to permanent teeth. Previous research has indicated that this factor may play a significant role in the accelerated and heightened progression of erosion and dental caries in the enamel of deciduous teeth [8].

### 4.1. Limitations

It is important to note that the study has a limitation due to the reduced number of analyzed samples. Further studies are needed to enhance understanding of primary and permanent teeth's structural and molecular features. It is worth noting that the outcomes of this study are based solely on in vitro experimentation, which may hinder their direct applicability to in vivo scenarios. As such, the findings must be interpreted and applied with caution, considering the experimental setup's inherent limitations. While our study highlights the intrinsic differences among dental tissues, the disparity in the oral scenarios, mineralization properties, and anatomical components among individuals could impact the actual tissue dynamics. While the sample size and selection references were rigorously framed, they might not fully represent the diversity of the population. It is important to conduct more research to bridge the differences between laboratory observations and actual-life clinical scenarios. Additionally, factors such as systemic condition, gender, and age can help us better understand the differences in the chemical makeup of dental tissues. We acknowledge these limitations to emphasize the relevance of our findings as positive contributions to the extensive panorama of dental research. By integrating our research findings with clinical observations, we can develop personalized dental interventions that are tailored to the specific needs of each patient.

## 5. Conclusion

The novel observations gained in this investigation affix futuristic data to the research literature, which can display a pertinent understanding of the analytical techniques imperative to assess the microstructure and the distinct elemental/chemical configuration across several variants of primary and permanent teeth hold significant applications in clinics and thereby patient care. By shedding light on the variations in mineralization, functional groups, and organic content within various dental structures, our study lays a favorable basis for continuing revolutionary investigations.

## Figures and Tables

**Table 1:** Summary statistics of parameters studied.

Categories	Group variables	*n*	Mean area	Standard deviation
Incipient enamel caries	Permanent Deciduous	15 15	245.21 259.14	85.21 81.72

Dentin destruction (water)	Permanent Deciduous	15 15	1680.5 3443.5	386.09 638.96

Sclerotic dentin (air)	Permanent Deciduous	15 15	2017.7 362.6	163.81 34.60

**Table 2:** Comparison of incipient enamel caries between deciduous and permanent teeth with coronal caries.

Categories	*t*	df	Mean difference (permanent deciduous)	Std. error difference	95% Confidence interval of the difference		*p* value
					Lower	Upper	
Incipient enamel lesions	−0.457	27.951	−13.93	30.49	−76.38	48.52	0.651

*Note:p* value less than 0.05 was considered to be statistically significant.

**Table 3:** Comparison of incipient enamel lesion at Dentino–Enamel junction between deciduous and permanent teeth with coronal caries.

Categories	Groups	Percentage (%)	Median	Interquartile range	*p* value
Incipient enamel lesion at DEJ	Permanent	20	212.98	172.4, 234.85	0.2
	Deciduous	20	110.03	104.97, 183.96	

*Note: p* value less than 0.05 was considered to be statistically significant.

**Table 4:** Comparison of dentin destruction and sclerotic dentin formation between deciduous and permanent teeth with coronal caries.

Categories	*t*	df	Mean difference (permanent deciduous)	Std. error difference	95% Confidence interval of the difference		*p* value
					Lower	Upper	
Dentine destruction (water)	−9.15	23.02	−1762.92	192.76	−2161.65	−1364.19	< 0.001^∗^
Sclerotic dentine (air)	38.28	28	1655.08	43.23	1566.52	1743.64	< 0.001^∗^

^∗^ *p* value less than 0.05 was considered to be statistically significant.

**Table 5:** Comparison of peritubular dentin destruction and bacterial concentration between deciduous and permanent teeth with coronal caries.

Categories	Grades	Permanent % (*n*)	Deciduous % (*n*)	*p* value
Peritubular dentine destruction	Mild	62% *n* = 5	0% *n* = 0	0.002^∗^
	Moderate	37% *n* = 3	44% *n* = 4	
	Severe	0% *n* = 0	55% *n* = 5	

Bacterial concentration	Mild	12% *n* = 1	44% *n* = 4	0.015^∗^
	Moderate	37% *n* = 3	11% *n* = 1	
	Severe	37% *n* = 3	0% *n* = 0	

^∗^ *p* value less than 0.05 was considered to be statistically significant.

## Data Availability

The data that support the findings of this study are available on request from the corresponding author. The data are not publicly available due to privacy or ethical restrictions.
